# Dynamic mechanism of GPCR-mediated β-arrestin: a potential therapeutic agent discovery of biased drug

**DOI:** 10.1038/s41392-022-01140-6

**Published:** 2022-08-12

**Authors:** Zheng Xu, Zhenhua Shao

**Affiliations:** Division of Nephrology and Kidney Research Institute, State Key Laboratory of Biotherapy and Cancer Center, West China Hospital, Sichuan University, 610041 Chengdu, Sichuan China

**Keywords:** Biophysics, Cell biology

Recently, a study published in *Cell* by Asher and colleagues, discovered the β-arrestin-1 (βarr-1) activation could be mediated by its C-terminal tail, the activation of targeted receptor and pattern of phosphorylation at the C-tail of receptor, based on a single molecule fluorescence resonance energy transfer imaging strategy.^1^

G protein-coupled receptors (GPCRs) are the largest known superfamily of signaling proteins in most mammalian, which regulate various biological processes, like metabolism, cell growth, differentiation and sensing signal transmitting such as vision, taste and pain. GPCRs convert stimuli from the extracellular to the cytoplasm through two classical signaling pathways, the G protein dependent pathway and the β-arrestin (βarr) dependent pathway. These signaling pathways regulate the internal downstream signal cascade that triggers several cellular physiological processes. Potency of the drug targeting GPCRs commonly relies on one of the pathways whereas the side effect comes up from the other.

Generally, the βarr pathway depends on the phosphorylation of the GPCR C-terminal or intracellular loop region by the GPCR kinases (GRKs), which is essential for the GPCR/βarr interaction. In addition to arresting G protein dependent signaling by desensitization and internalization, βarrs are delineated to promote different signals in response to the GPCRs, the phosphorylation of MAP kinases ERK1/2 as a case.^2^ Structural studies of individual βarr and the βarr in complex with GPCRs depict the conformational rearrangement of βarr between inactive and active states, yet the C-terminal tail of βarr, associated with the binding of the downstream cascade proteins like clathrin, adaptin and ERK2, is either truncated or invisible in these structures.^1,2^ The dynamic and kinetic of how βarr interact with GPCRs and βarr tail release in the active βarr are essential for understanding βarr-mediated signaling and guiding βarr biased drug designing.

To elucidating these issues, Asher and colleagues primarily explored the dynamic and conformation of βarr1 tail in the basal (inactive) state using molecular dynamics (MD) simulations. Based on the simulations, authors designed a βarr1 construct suitable for the smFRET (single-molecule Fluorescence Resonance Energy Transfer) imaging to distinguish the status whether βarr1 tail released (resembles active state) from the N-domain groove (resembles inactive state). The presence of phosphorylated human V2 vasopressin receptor C terminal poly peptide (V_2_Rpp) but not the non-phosphorylated poly peptide (V_2_Rnp) induced a reversible transition of FRET efficiency, implicating that the phosphorylation of the peptide V_2_Rpp is essential for the activation of βarr1. In addition, a naturally occurred glycosaminoglycan, named heparin, is used to activate βarr and can also induce the FRET efficiency alternation, which is distinct from that induced by V_2_Rpp. This observation indicated that the C-tail of the activated βarr1 could exhibit at least two distinct conformations, further implicating the dynamic and versatile of activated βarr1 in response to the downstream signaling.

Moreover, the authors examined the interaction of the intact receptor to the βarr1 based on smFRET. They used the β_2_ adrenergic receptor chimera with a C-tail substitution by the V_2_R C-tail (referred as β_2_V_2_R hereafter), which is auspicious for the investigation with βarr1 interaction and activation. The result shows that combining the fully phosphorylated β_2_V_2_R C-terminal tail and the activation of the β_2_V_2_R by the full agonist epinephrine, could induce the βarr1 tail releasing and activation. Moreover, introducing positive allosteric modulator (PAM) could elevate the extent of receptor activation, thus increasing the activation level of βarr1 compared with the one only activated by agonist alone. The authors hypothesized that the C-tail of β_2_V_2_R was obstructed to contact with βarr1 in the inactive receptor, and convincingly consolidated it by a serious of antibody based competitive binding experiment.^1^

Finally, the authors examined the hypothesis-driven studies for their finding in vivo. Due to the reason that the phosphorylation of GPCR by GRKs is generally mediated by the agonist stimulation for receptor, the authors knocked out the four GRKs (GRK2/3/5/6) in HEK293 cells (HEK_GRK-KO_), blocking the phosphorylation pattern in the agonist activating condition. They found that βarr2 was unable to be recruited to the plasma membrane by activated wild-type β_2_AR, but significantly accumulated on the membrane expressing C-terminal truncated β_2_AR-365tr, based on the bystander bioluminescence resonance energy transfer (BRET) assay. These evidences indicate that the removal of the β_2_AR C-terminal tail results in more efficiently recruiting βarr2 and leads to the core engagement of the βarr2 with receptor. These results indicating that the phosphorylation of receptor is insufficient for the GPCR/βarr interaction and βarr activation, implicating that the βarr-biased signaling is relevant for the activation of GPCR and the phosphorylation.

**Fig. 1:**
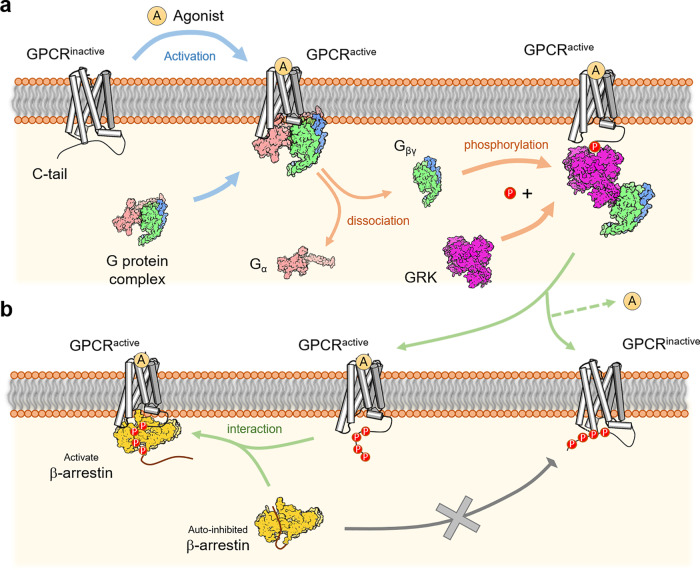
Schematic illustration of the activating procedure of GPCR as well as the agonist-induced signaling event. **a** (Up) The heterotrimeric G protein complex interacts with the activated GPCRs, therefore triggers the dissociation of the Gα and Gβγ subunit, where the latter recruits GRKs to phosphorylate the C-terminal tail of GPCR. **b** (Down) The βarr tends to interact with activated and phosphorylated GPCRs than the phosphorylation-only GPCRs

Generally, activated GPCR could engage G protein signals, which release its G_βγ_ subunit and recruit cytoplasm GRKs to phosphorylate the GPCR, whereupon promoting βarr recruitment for the GPCR desensitization and internalization (Fig. 1). Both G protein signaling and βarr signaling are essential and contribute to the cell signal transduction and specific biophysical process. For the opioid receptors, for example, the G protein (G_i_) signal pathway is proposed for analgesia, whereas the βarr dependent signal is response for the adverse effect, like addiction and tolerance;^3^ whereas both G protein and βarr dependent signaling are required for anti-inflammatory effect in response for PTH1R^4^ and the G protein signaling of the Dopamine D1 receptor (DRD1) play roles in inhibiting inflammatory reactions, maintaining cardiovascular as well as kidney homeostasis, nevertheless desensitized through βarr dependent pathway.^5^

Previous studies reveal the phosphorylated GPCRs is pivotal for the recruitment of βarr. The resolved complex structures of the GPCR and βarr displayed the binding snapshot due to the flexibility of the tail-only engagement.^1^

Accuracy and low side effects are essential criterion for drug designing, to clarify the activating process of βarr in response to GPCR step by step helps to do so. Asher and colleagues shed light on the dynamic process of βarr upon interacting with GPCRs, and illuminated that the GPCR/βarr interaction depended on the pattern of phosphorylation within the receptor tail and the extent of receptor activation. Meanwhile, the smFRET strategy developed in this article can also be utilized for the evaluation of novel designed drugs in the βarr bisased signaling, for instance, desensitization by the recruitment of clathrin by the released C-terminal tail of βarr. In addition, the βarr biased signaling is implicated in the specific disease therapy by its unique downstream signals. this study demonstrates the potency of the βarr biased agonist is crucial for the treatment of the βarr dependent pathway related disease, which ultimately assists the identification and development of biased drugs.
